# Computational fluid dynamics and shape analysis enhance aneurysm rupture risk stratification

**DOI:** 10.1007/s11548-024-03289-7

**Published:** 2024-11-17

**Authors:** Ivan Benemerito, Frederick Ewbank, Andrew Narracott, Maria-Cruz Villa-Uriol, Ana Paula Narata, Umang Patel, Diederik Bulters, Alberto Marzo

**Affiliations:** 1 INSIGNEO Institute for in Silico Medicine, University of Sheffield, Sheffield, UK; 2 Department of Mechanical Engineering, University of Sheffield, Sheffield, UK; 3 Department of Neurosurgery, University Hospital Southampton, Southampton, UK; 4 Division of Clinical Medicine, University of Sheffield, Sheffield, UK; 5 Department of Computer Science, University of Sheffield, Sheffield, UK; 6 Department of Neuroradiology, University Hospital Southampton, Southampton, UK; 7 Department of Neurosurgery, Oxford University Hospital NHS Foundation Trust, Oxford, UK

**Keywords:** Aneurysm, Fluid dynamics, PHASES, Logistic regression, Risk factors, Shape

## Abstract

**Purpose:**

Accurately quantifying the rupture risk of unruptured intracranial aneurysms (UIAs) is crucial for guiding treatment decisions and remains an unmet clinical challenge. Computational Flow Dynamics and morphological measurements have been shown to differ between ruptured and unruptured aneurysms. It is not clear if these provide any additional information above routinely available clinical observations or not. Therefore, this study investigates whether incorporating image-derived features into the established PHASES score can improve the classification of aneurysm rupture status.

**Methods:**

A cross-sectional dataset of 170 patients (78 with ruptured aneurysm) was used. Computational fluid dynamics (CFD) and shape analysis were performed on patients’ images to extract additional features. These derived features were combined with PHASES variables to develop five ridge constrained logistic regression models for classifying the aneurysm rupture status. Correlation analysis and principal component analysis were employed for image-derived feature reduction. The dataset was split into training and validation subsets, and a ten-fold cross validation strategy with grid search optimisation and bootstrap resampling was adopted for determining the models’ coefficients. Models’ performances were evaluated using the area under the receiver operating characteristic curve (AUC).

**Results:**

The logistic regression model based solely on PHASES achieved AUC of 0.63. All models incorporating derived features from CFD and shape analysis demonstrated improved performance, reaching an AUC of 0.71. Non-sphericity index (shape variable) and maximum oscillatory shear index (CFD variable) were the strongest predictors of a ruptured status.

**Conclusion:**

This study demonstrates the benefits of integrating image-based fluid dynamics and shape analysis with clinical data for improving the classification accuracy of aneurysm rupture status. Further evaluation using longitudinal data is needed to assess the potential for clinical integration.

**Supplementary Information:**

The online version contains supplementary material available at 10.1007/s11548-024-03289-7.

## Introduction

The management of patients with unruptured intracranial aneurysms (UIA) remains a clinical challenge. While 3% of adults have one, only a small percentage of these rupture [1]. When they do rupture, they cause subarachnoid haemorrhage, which results in significant morbidity and mortality [2].

Currently, the only options for prophylactic treatment are endovascular and surgical interventions [2]. Despite advances in these approaches, they remain associated with complication rates ranging from 3 to 10% [3, 4]. Subsequently, in clinical practice, the decision on whether to treat an aneurysm relies on comparing the perceived risk of its rupture against the risks associated with its treatment. In recent years, a number of natural history studies have investigated factors associated with aneurysm rupture. The standard risk stratification model for estimating the risk of rupture is the PHASES score [5], which uses six clinical variables (ethnicity, hypertension, age, aneurysm size, aneurysm location, history of previous SAH) to estimate the five-year risk. While this scoring system achieved an AUC (Area Under the Curve, where the curve is the Receiver Operating Characteristic, ROC) of 0.82 on its original dataset, its performance deteriorates when applied to external data [6–8].

Although the best available model, the PHASES score remains a crude assessment of the risk of rupture and there remains a need for more detailed and personalised risk estimates. Angiographic imaging and derived haemodynamic and morphological data hold vast potential. In recent years, the development of computational fluid dynamics (CFD) has provided insight into aneurysm rupture, with factors like wall shear stress [9], oscillatory shear index [10] and residence time [11] already explored. This has led to the understanding that aneurysms with irregular shapes (e.g. high aspect ratio [12] or presence of bulges [13]) or that exhibit complex flow patterns [14] are more prone to rupture, and identification of novel risk factors derived from shape and haemodynamic analyses. These factors have been integrated into several rupture classification models [14–17], although none has been implemented in clinical practice. One reason for this is that CFD and morphology models have not been tested to see if they add any predictive value over and above routinely available clinical variables. To improve their clinical uptake, it is also necessary to reduce their lengthy processing, feature-extraction, and evaluation times, to show that the feature extraction operation can be performed repeatably and reliably, and to show that the adoption of computer-derived data is beneficial in a clinical context.

This study’s first aim is to evaluate whether haemodynamic and morphological data generated within the pipeline developed as part of the European project @neurIST [18], can improve the distinction between ruptured and unruptured aneurysms compared to the PHASES score. Its second aim is to identify which image-derived features of aneurysm shape and flow most strongly correlate with rupture status in order to optimise the feature set and facilitating integration of computational risk models with existing clinical workflows.

## Methods

### Patient population

Patient data was collected within the European Union funded @neurIST project (FP6-IST). In this study patients were recruited from seven clinical centres across Europe (UK, Switzerland, Hungary, Netherlands, Spain, and Ireland). Ethical approval and patient consent were obtained for all patients according to local ethics and data usage regulations. All images and patient data were processed exclusively within the @neurIST project.

The @neurIST project recruited over 1400 participants. However, only those with both 3D Rotational Angiographies (3DRA) and complete PHASES data were included for analysis and processed to obtain patient-specific morphological and haemodynamic data. This resulted in a final study population of 170 patients, divided into 78 with ruptured aneurysms and 92 with unruptured aneurysms.

The representativeness of the dataset was tested by comparing the distributions of demographic and aneurysm characteristics with those in the PHASES study, which also includes the UCAS and ISUIA studies and amounted to 8382 participants [5]. These are shown in Table 1.

**Table 1:** Comparison of demographic and aneurysm characteristics between PHASES and @neurIST datasets

	PHASES		@neurIST dataset	
	Rupture (*n* = 220) (%)	No rupture (*n* = 8162) (%)	Rupture (*n* = 78) (%)	No rupture (*n* = 92) (%)
**Patient characteristics**				
*Women*	74	68	72	74
*Age*				
< 40 years	12	5	12	9
40–49 years	11	13	33	33
50–59 years	16	28	38	39
60–69 years	25	33	12	16
≥ 70 years	37	24	8	4
*Hypertension*	53	44	27	44
*Previous SAH*	23	13	6	4
**Aneurysm characteristics**				
*Size at the time of detection*				
< 5.0 mm	26	48	70	35
5.0–6.9 mm	13	26	20	31
7.0–9.9 mm	18	15	21	27
10.0–19.9 mm	29	11	7	4
≥ 20 mm	17	2	3	0
*Location*				
ICA	38	38	18	42
MCA	25	34	27	23
ACA/PCA	38	29	56	36

Unlike the dataset from the PHASES study, the @neurIST dataset presents a relatively balanced split between ruptured and unruptured cases. Furthermore, the distribution of patients between ruptured and unruptured cases differs for the two datasets for key features such as hypertension and previous SAH, as well as for all age groups except those younger than 40 years. Because of the different distributions between PHASES and @neurIST, as well as the fact that the PHASES score was developed from longitudinal data, while our dataset is of cross-sectional nature, in building the logistic regression models we decided not to use the aggregate score of the six PHASES variables as originally developed in [5], but rather treated the PHASES variables as individual and independent features. Further details are provided in the Additional Material.

### Morphological and haemodynamics analysis

The @aneurIST toolchain, described in detail in [19], was employed to segment all 3DRA images, allowing for the extraction of the specific geometry for each individual aneurysm. Segmented geometries were meshed to run a computational fluid dynamics (CFD) analysis of the blood flow within the aneurysm and in the surrounding regions. A full cardiac cycle was simulated: inlet conditions were imposed as a flow-rate waveform, whereas outlet conditions were represented using a pressure waveform. Flow-rate and pressure waveforms were computed with a generic whole body 1D circulation model set-up within the @neurIST toolchain [20].

Medical images and segmented geometries were processed to extract the patient-specific morphological features of the aneurysm. While the toolchain aimed for automation, the evaluation of certain morphological variables such as the location of the aneurysm neck (which must be identified through manual landmarking) and the aspect ratio required human intervention. Guidelines were provided to ensure consistency of these subjective variables. Similarly, relevant haemodynamic variables were determined by post-processing the results of the CFD simulations. This yielded the morphological (shape) and haemodynamic (CFD) variables shown in Table 2. As explained in the next section, only a subset of the variables shown in Table 2 are included in the final model.

**Table 2:** Complete list of morphological and haemodynamics variables with their definition, before the highly correlated features are removed from the analysis

Feature	Definition	Type	Units	Assessment
AR	Aspect ratio	Shape	Adimensional	Manual
NWidth	Diameter of aneurysm neck	Shape	m	Manual
Surface	Aneurysm surface	Shape	m^2^	Automatic
NSurf	Surface area of aneurysm neck	Shape	m^2^	Automatic
Vol	Aneurysm volume	Shape	m^3^	Automatic
NSI	Non-sphericity index	Shape	Adimensional	Automatic
AbsHighWSSAreaAvg	Area of high WSS, average over time within aneurysm	CFD	m^2^	Automatic
AbsLowWSSAreaAvg	Time average of low WSS area within aneurysm	CFD	m^2^	Automatic
RelHighWSSAvg	Percentage of high WSS area, averaged over time within aneurysm	CFD	Adimensional	Automatic
RelLowWSSAreaAvg	Percentage of aneurysm area with low WSS, averaged over time	CFD	Adimensional	Automatic
AbsHighWSSAreaPeak	Area of high WSS at peak systole within aneurysm	CFD	m^2^	Automatic
RelHighWSSPeak	Percentage of aneurysm area with high WSS at peak systole	CFD	Adimensional	Automatic
MaxWSSPeak	Maximum WSS at peak systole	CFD	Pa	Automatic
AbsHighOSIArea	Area of elevated OSI within aneurysm	CFD	m^2^	Automatic
RelHighOSIArea	Percentage of area with high OSI within aneurysm	CFD	Adimensional	Automatic
MaxOSI	Maximum oscillatory shear index within aneurysm	CFD	Pa	Automatic
AbsHighPAreaPeak	Area of high pressure at peak systole within aneurysm	CFD	m^2^	Automatic
RelHighPAreaPeak	Percentage of aneurysm surface with high pressure at peak systole	CFD	Adimensional	Automatic
MaxPPeak	Maximum pressure at peak systole within aneurysm	CFD	Pa	Automatic
AvgVAvg	Average velocity inside aneurysm, averaged over time	CFD	m/s	Automatic
AvgVPeak	Average velocity inside aneurysm at peak systole	CFD	m/s	Automatic
MaxVAvg	Time average of maximum velocity inside the aneurysm	CFD	m/s	Automatic
MaxVPeak	Maximum velocity in aneurysm at peak systole	CFD	m/s	Automatic
MaxVNPeak	Maximum velocity in aneurysm neck at peak systole	CFD	m/s	Automatic
MaxFluxNeck	Maximum flux through aneurysm neck	CFD	kg*m/s	Automatic
MomFluxPeak	Momentum flux through aneurysm neck at peak systole	CFD	kg*m/s	Automatic
AbsInfluxAreaPeak	Area of influx flow into the aneurysm at peak systole	CFD	m^2^	Automatic
RelInfluxAreaPeak	Percentage of neck area with influx flow into aneurysm at peak systole	CFD	Adimensional	Automatic
VisDis	Viscous dissipation within aneurysm	CFD	W	Automatic

### Classification models

To assess the potential of image-based computed data in improving the classification of aneurysm rupture compared to using PHASES alone, five logistic regression models were constructed (Table 3). The reference model (*P*) included only PHASES variables. The remaining models evaluated the contribution of additional data:

- Model PC combined PHASES with CFD-derived features
- Model PS combined PHASES with shape-derived features
- Model PCS incorporated both CFD and shape features along with PHASES variables.
- Model CS solely relied on CFD and shape features, excluding PHASES.

**Table 3:** Number of variables after feature reduction, including the dummy variables used for the categorical features

Feature group	Initial number of features	Number of features when threshold = 0.7	Number of features when threshold = 0.8	Number of features when threshold = 0.9
P	6	6	6	6
PC	30	20	23	27
PS	12	9	10	10
PCS	36	23	27	31
CS	30	17	21	25

### Feature reduction: correlation coefficient and principal component analysis

To prevent issues arising from multicollinearity of the input variables, we reduced the dimensionality of the C (CFD-derived) and S (shape-derived) features before training the classification model. We aimed at retaining all the PHASES variables in all models. However, we excluded ethnicity due to a lack of variability as the dataset included no patients from Finland or Japan. Additionally, the size of the aneurysm (AneuDepth) obtained from the @neurIST toolchain was treated as a PHASES variable, not as an S variable.

Lasso optimisation has been previously used in the literature for automatic feature reduction of logistic regression models [15, 21]. However, preliminary investigation of the dataset using lasso regression resulted in a feature pool that did not include any PHASES variables, thus violating our initial aim. Attempts to force the inclusion of PHASES variables within the final model by modifying the penalty term in the cost function proved not effective. Thus, we performed feature reduction using two distinct methods [22, 23], and then used the reduced feature set for ridge logistic regression [24]:

I. Pearson correlation coefficient to identify features with high correlation, potentially indicating redundancy.
II. Principal Component Analysis (PCA) to create a new set of uncorrelated features that capture most of the information in the original data.

For the correlation-based method, we removed features with Pearson correlation coefficients exceeding a specific threshold, ranging from 0.7 to 0.9, and that reached significance level (*p* < 0.01). The resulting models (P, PS, PC, PCS, and CS) and the number of features used in each are shown in Table 3.

For the PCA-based method, we computed the principal components of the CFD and shape variables separately, and then combined them with the PHASES variables to obtain the PCA-derived P, PS, PC, PCS, CS models. PCA analysis was performed using the Python3 package Sklearn, version 1.3 [25].

### Logistic regression models

For both correlation-based and PCA-based models, the same statistical modelling procedure was used. All the numerical variables were treated as continuous variables and standardised by subtracting their standard deviation and dividing by their mean values. Categorical variables from PHASES were included as dummy variables. We used ridge logistic regression [24], implemented in Sklearn, for classifying ruptured and unruptured aneurysms [14, 15, 21]. Logistic regression computes the probability $p({\varvec{x}})$ that an event occurs, in this case an aneurysm belonging to the ruptured group, given a set of $n$ parameters ${\varvec{x}}=\left\{{x}_{1},\cdots ,{x}_{n}\right\}$ and the set of coefficients ${\varvec{\beta}}=\{{\beta }_{0},{\beta }_{1},\cdots , {\beta }_{n}\}$. This is done through the logistic regression equation:

1 $$ p\left( x \right) = \frac{1}{{1 + e^{{ - \left( {\beta_{0} + \beta_{1} x_{1} + \cdots + \beta_{n} x_{n} } \right)}} }} $$

Each parameter ${x}_{i}$ represent a PHASES, CFD or shape variable. The coefficients ${\beta }_{i}$ indicate the weight of the parameter ${x}_{i}$ in determining the probability $p({\varvec{x}})$ and are computed through model fitting. For the purpose of model fitting, we used eighty percent of the dataset for training and the remaining twenty percent for testing. We randomised the splitting by performing ten-fold cross validation. Within each fold we optimised the ridge regularisation parameters and the model coefficients through a grid search algorithm under the constraint that the AUC of the resulting ROC was maximised. This procedure of randomised splitting and optimisation was repeated with 500 iterations of boostrap resampling to compute the confidence intervals of the model coefficients [15]. The best performing model, that is the model that showed the highest AUC during training, was then evaluated on the test dataset. Since we designed the logistic regression to predict the probability that patients belong to the ruptured group, a positive coefficient ${\beta }_{i}$ means that the feature ${x}_{i}$ is associated with membership to the ruptured group.

## Results

### Feature reduction: Pearson’s correlation

As shown in Table 3, the number of variables retained in the model changed with the correlation threshold: an increase in the threshold reduced the number of variables that were filtered out, thus leading to models that require more parameters. The list of features retained in each group for a correlation threshold 0.7 are shown in Table 5.

### Feature reduction: PCA

**Fig. 1:**
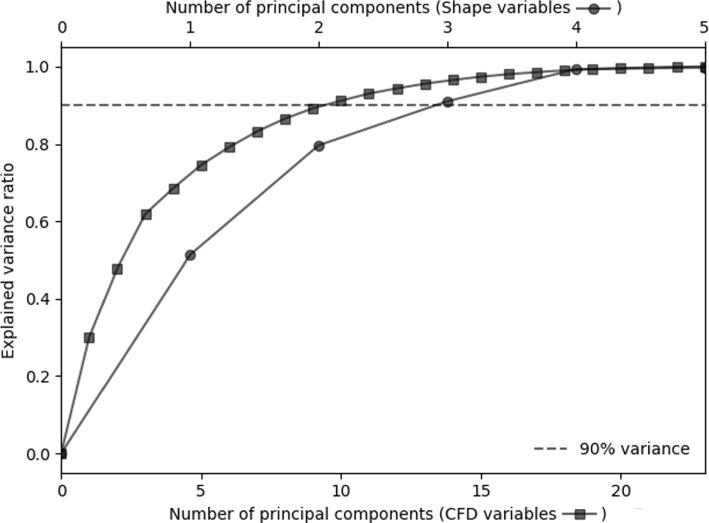
Percentage of explained variance as a function of the number of principal components for haemodynamics and morphological features

The amount of variance explained by the principal components of CFD-derived and morphological features is shown in Fig. 1. In what follows, we will present the results of the logistic regression models built with ten components for CFD variables and three for shape features, that were able to explain 90% of the variance of the original features.

### Classification models

**Fig. 2:**
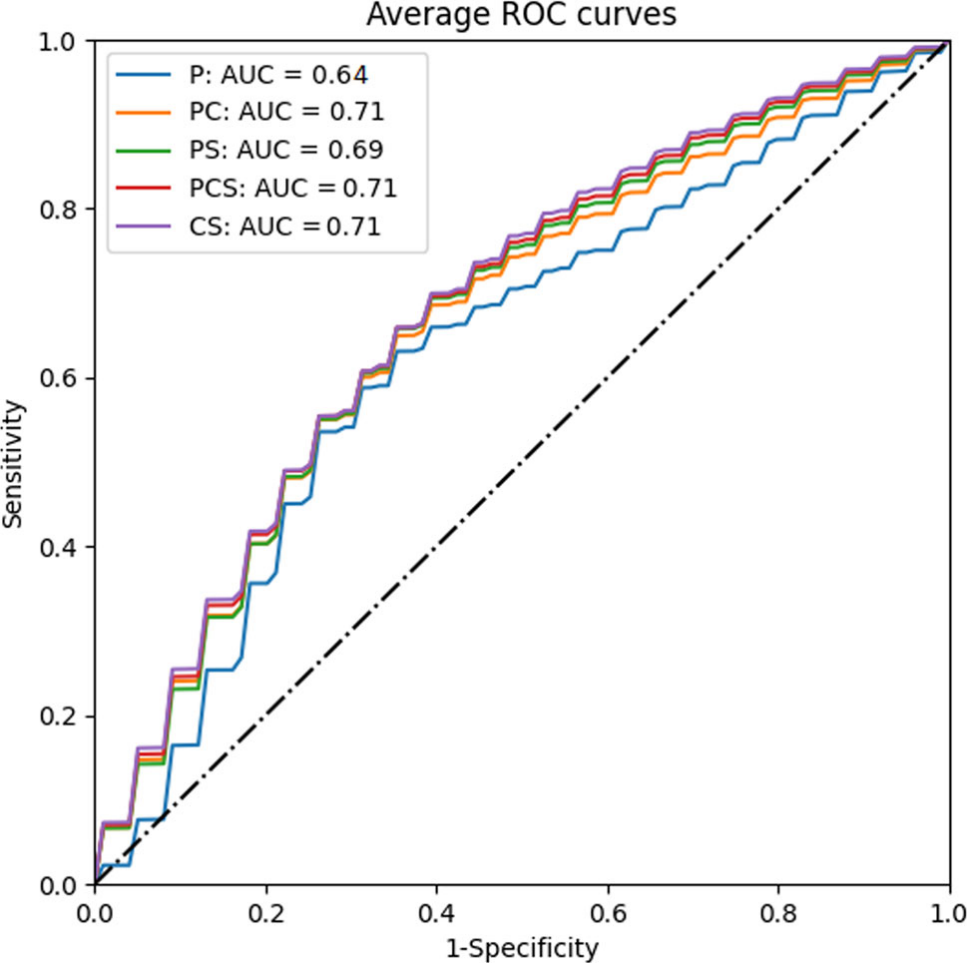
Average ROC curves for the five correlation-based logistic regression models. The ROC curves for each group are obtained using the variables that are retained after the correlation-based feature reduction. The feature for each group are listed in the corresponding column of Table 3

Changing the correlation threshold in the feature reduction approach using Pearson’s correlation had only a minor effect, thus we show in Table 4 only the results obtained when such threshold was set to 0.7. When the correlation-based feature reduction method is adopted, using only PHASES variables produced the lowest AUC values, with mean ± standard deviation AUC = 0.636 ± 0.084. The inclusion of shape data increased the AUC to 69.2 ± 7.9. Inclusion of CFD variables also improved prediction, with AUC = 0.71 ± 0.085 for the PC group. Further addition of both CFD and shape variables did not further improve classification in the PCS model (AUC = 0.709 ± 0.084). The use of derived data alone (group CS) yielded an AUC = 0.709 ± 0.084. The corresponding average ROC curves are shown in Fig. 2.

**Table 4:** Performance of the logistic regression models. Since PHASES variables are never removed, results for correlation-based and PCA-based logistic regression models are the same

Feature group	Correlation based	PCA based
P	0.64 ± 0.08	
PS	0.69 ± 0.08	0.66 ± 0.09
PC	0.71 ± 0.08	0.69 ± 0.08
PCS	0.71 ± 0.08	0.68 ± 0.08
CS	0.71 ± 0.08	0.69 ± 0.08

Results from using logistic regression models after PCA-based model reduction are consistent and show similar performance.

Table 5 presents the average value and 95% confidence intervals for the coefficients of the best logistic regression models for each group, after the feature reduction with correlation threshold 0.7. As explained above, positive sign of the coefficient means that the corresponding feature is associated with ruptured status. Most of the features tend to be associated with only state, either ruptured or unruptured. The only exceptions are represented by Age, AneuDepth and RelLowWSSAreaAvg. Results from logistic regression on the P group identify both Age and AneuDepth as indicative of rupture status. They are however evaluated as characteristic of unruptured status in groups PC, PS and PCS. Similar behaviour is observed for RelLowWSSAreaAvg, which appears to be associated with rupture in PC and with unruptured in PCS and CS. NSI and MaxOSI are the variables that are most strongly associated with rupture and show narrow 95% confidence intervals. This is true for all the groups where these variables are included, in which they consistently rank high. In agreement with this, patients where large areas of the aneurysm are subject to high OSI are more likely to suffer a rupture (variable RelHighOSIArea). Likewise, rupture is associated with low wall shear stresses (variables AbsHighWSSAreaPeak, AbsLowWSSAreaAvg), high values of velocity inside the aneurysm region and high viscous dissipation. Large high-pressure areas and volumes are indicative of unruptured aneurysms.

**Table 5:** coefficients of the final logistic regression models (correlation threshold: 0.7) of the five groups with the 95% confidence interval reported in brackets

Feature	P	PC	PS	PCS	CS
Age *	0.009	− 0.013	− 0.023	− 0.022	
	[0.002; 0.017]	[− 0.015; − 0.01]	[− 0.031; − 0.016]	[− 0.027; − 0.018]	
AneuDepth *	0.031	− 0.025	− 0.332	− 0.136	
	[0.021; 0.04]	[− 0.033; − 0.017]	[− 0.349; − 0.315]	[− 0.154; − 0.118]	
Location_MCA +	0.933	0.037	0.852	0.11	
	[0.898; 0.968]	[0.026; 0.048]	[0.826; 0.879]	[0.094; 0.126]	
Location_PCOM +	1.107	0.088	0.904	0.203	
	[1.074; 1.141]	[0.075; 0.102]	[0.88; 0.927]	[0.18; 0.226]	
EarlierSAH_Yes +	0.421	**0.001**	0.326	0.022	
	[0.362; 0.479]	**[− 0.004; 0.007]**	[0.28; 0.371]	[0.009; 0.035]	
Hypertension_Yes** -**	− 0.73	− 0.084	− 0.569	− 0.155	
	[− 0.749; − 0.71]	[− 0.095; − 0.074]	[− 0.586; − 0.553]	[− 0.169; − 0.14]	
NWidth +			0.284	0.189	0.132
			[0.272; 0.295]	[0.164; 0.214]	[0.113; 0.151]
NSI +			0.707	0.248	0.214
			[0.695; 0.72]	[0.23; 0.266]	[0.2; 0.229]
Vol -			− 0.162	− 0.123	− 0.148
			[− 0.18; − 0.143]	[− 0.136; − 0.109]	[− 0.169; − 0.126]
AbsHighPAreaPeak -		− 0.143		− 0.281	− 0.255
		[− 0.155; − 0.13]		[− 0.305; − 0.256]	[− 0.279; − 0.231]
AbsHighWSSAreaPeak -		− 0.017		− 0.058	− 0.052
		[− 0.02; − 0.014]		[− 0.066; − 0.05]	[− 0.059; − 0.044]
AbsHighWSSAreaAvg -		− 0.079		− 0.17	− 0.164
		[− 0.087; − 0.071]		[− 0.185; − 0.155]	[− 0.18; − 0.148]
AbsLowWSSAreaAvg +		0.082		0.157	0.144
		[0.073; 0.09]		[0.143; 0.171]	[0.128; 0.161]
AvgVPeak -		− 0.023		− 0.029	− 0.022
		[− 0.027; − 0.018]		[− 0.039; − 0.02]	[− 0.031; − 0.012]
MaxOSI +		0.163		0.24	0.22
		[0.15; 0.175]		[0.226; 0.254]	[0.206; 0.233]
MaxPPeak -		− 0.01		− 0.027	− 0.017
		[− 0.014; − 0.005]		[− 0.035; − 0.018]	[− 0.026; − 0.009]
MaxVPeak +		0.046		0.057	0.044
		[0.041; 0.052]		[0.048; 0.066]	[0.034; 0.054]
RelHighOSIArea +		0.053		0.066	0.088
		[0.049; 0.056]		[0.058; 0.074]	[0.078; 0.097]
RelHighPAreaPeak -		− 0.086		− 0.05	− 0.045
		[− 0.092; − 0.081]		[− 0.06; − 0.04]	[− 0.056; − 0.035]
RelHighWSSPeak -		− 0.055		− 0.023	− 0.014
		[− 0.06; − 0.05]		[− 0.029; − 0.016]	[− 0.021; − 0.006]
RelLowWSSAreaAvg *		0.023		− 0.018	− 0.021
		[0.017; 0.029]		[− 0.03; − 0.007]	[− 0.034; − 0.007]
RelInfluxAreaPeak -		− 0.078		− 0.098	− 0.112
		[− 0.081; − 0.074]		[− 0.104; − 0.093]	[− 0.118; − 0.106]
VisDis +		0.064		0.166	0.152
		[0.052; 0.076]		[0.142; 0.19]	[0.129; 0.175]
Intercept -	− 0.513	− 0.025	− 0.451	− 0.078	− 0.02
	[− 0.534; − 0.492]	[− 0.032; − 0.019]	[− 0.467; − 0.436]	[− 0.089; − 0.066]	[− 0.023; − 0.018]

The table contains only the variables that are retained in the models after the feature reduction stage. Positive coefficients mean that the feature is associated with rupture status and are marked with a plus sign ( +). Negative coefficients mean that the feature is associated with unruptured status and are marked with a minus sign (−). Features that are associated with ruptured or unruptured status depending on the group in which they are included are marked with an asterisk (*). Bold coefficients indicate that the confidence interval includes the origin

In accordance with what was observed before, the PCA-derived models show that haemodynamic or morphological data improve the predictive power of the logistic regression models. As PCA features are not interpretable and do not bear clinical relevance, their coefficients are not reported in the manuscript.

## Discussion

This study assessed five logistic regression models constructed from different combinations of clinical, morphological (shape), and haemodynamic data for classifying aneurysms’ rupture status. Previous studies have identified several morphological and haemodynamic factors which show statistical association with aneurysm rupture, and have shown that they can be used to classify ruptured and unruptured cases [7, 14,  and 26]. However, they have not assessed whether the addition of these can improve established risk prediction models with respect to clinically established protocols. Our results show that, when compared to the PHASES score alone, the addition of either morphological or haemodynamic data individually improved classification accuracy. The addition of both morphological and haemodynamic data did not cause any further improvement.

Throughout our study, we also showed how through statistical approaches on a relatively large dataset we can reduce the number of image-derived features to those that demonstrate strongest association. This has been extensively debated through the scientific community [27–29], which recognises the importance of using robust statistical approaches and considerations when evaluating new potential image-derived features. This helps to avoid the proliferation of potentially confounding and misleading features, often arising from small, single-centre studies with limited populations. Such features may lack statistical significance and hinder generalisability of results.

Our results show that haemodynamic analysis can effectively improve the separation between ruptured and unruptured cases. We have found that high OSI and low WSS are indicative of rupture, which is in accordance with previous observations [9, 26,  and 30]. In the final set of retained variables, WSS does not directly appear through its values, but rather in terms of the area of the region where its values are high or low. This derives from the feature-reduction stage of the analysis, where we observed that MaxWSSPeak showed a high correlation (*r* = 0.88, *r* < 0.001) with MaxVPeak and was thus removed from the pool. The extension of aneurysm surface area exposed to low WSS (AbsLowWSSAreaAvg) is positively associated with rupture. This implies that, in case of rupture, large parts of the aneurysm are exposed to low WSS, a condition that has been identified as responsible for initiation, growth and rupture because of their role in endothelial remodelling [10, 31]. The association of WSS with rupture, however, is controversial and there exist studies that have linked rupture with high WSS [32]. This inconsistency can be partially explained by the observations in [33], where the authors hypothesised that aneurysms of different size fail because of various combinations of high/low WSS and oscillatory shear index (OSI). The OSI is a quantity derived from WSS that quantifies the oscillatory behaviour of the WSS vector along the cardiac cycle [34]. High OSI values are indicative of complex and dynamic flow patterns, vortices and recirculation regions within the aneurysm which ultimately induce low WSS and lower residence time [35]. Our models identify these conditions as associated with rupture status, coherently with results reported in [9, 36].

Complex haemodynamics is often induced by irregular shapes and geometries. NSI and AR are generally recognised as aggregated metrics for describing aneurysms’ shape, with higher values describing shapes that differ from an ideal sphere [12, 37]. Deviations from spherical shape yield uneven distributions of wall stress which enhance aneurysm instability and favour rupture [12]. NSI higher than 0.2 have shown significant discriminatory power in multiple studies [12, 37]. Our cohort of ruptured patients presented NSI = 0.198 ± 0.07, and that feature ranked as highly influential in our analysis. AR was part of the initial set of variables but, despite being widely acknowledged as a risk factor for aneurysm rupture [12, 37], it was excluded from the final analysis. This was because of its correlation with the PHASES variable AneuDepth (*r* = 0.79, *p* < 0.001) and, by design, we decided to retain all the PHASES variables. Studies [5, 38] observed that patients with ruptured aneurysms showed higher values of AneuDepth. This association is confirmed in our analysis when only the PHASES variables are used as predictors but show an opposite direction when derived data are included. Previous studies [39, 40] have shown that narrow necks and larger volumes can induce blood stagnation within the aneurysm, thus creating haemodynamic conditions favourable for rupture. We did not observe this phenomenon in our results, and we ascribe it to the features of our cohort, where patients with large volumes and narrow necks tend to belong to the unruptured group. Other researchers have reported conclusions similar to ours [15, 41].

Despite this study showing the improved classification of aneurysm rupture with the addition of CFD and morphological derived factors, there remain several limitations. In terms of the CFD, the boundary conditions were obtained from generic 1D models of the brain circulations, while personalised models might offer a more precise representation of the boundary conditions. In terms of modelling, we did not include in our analysis variables derived from patients’ lifestyle such as smoking and diet, or related to familial history of ruptured aneurysms and other cardiovascular pathologies. These are very well-established risk factors and regularly taken into consideration by doctors when evaluating possible treatment strategies [42]. However, they are not included among the PHASES variables and, since our study aims at evaluating the potential of CFD and shape analysis alongside the use of PHASES variables, we ultimately decided to exclude them from our model.

In terms of the population, our study is similar to previous studies [10, 26], and suffers from limited sample size and the cross-sectional nature of the dataset. The cross-sectional design of this study can explain a number of findings. It is not clear what is the effect of rupture on aneurysm shape and volume. Some authors report increases or while others report no modification [43, 44] in small-cohort studies. Our models identify smaller aneurysms (both in terms of Vol and AneuDepth) as predictive of unruptured status: however, without longitudinal observations to track their evolution, it is not possible to reach definitive conclusions on their role. Additionally, age showed an ambiguous effect in our models with elderly patients being more likely to rupture when exclusively considering the PHASES features, and less prone to rupture when shape and haemodynamics are taken into account. While there are conflicting views regarding the role of age [45], elderly patients are more likely to have a diagnosis of an UIA and this will influence the structure of a dataset. Regarding hypertension, it was not associated with rupture. Our dataset did not include any actual blood pressure values, however. It is therefore possible that more patients with ruptured aneurysms had undiagnosed hypertension, whereas more patients with unruptured aneurysms had their blood pressure controlled on antihypertensive agents. Furthermore, only 27% of the patients in the ruptured group suffered from hypertension, which likely introduced a bias. Our dataset also included a similar number of ruptured and unruptured cases and did not represent the distribution of aneurysms in the general populations. This is an issue common to most currently available studies [10, 13, 15]. The dataset was also cross sectional and was used to develop a classifier capable of distinguishing someone with a ruptured aneurysm from someone with an unruptured aneurysm. The dataset did not allow us to assess if it could predict which unruptured aneurysms would go on to rupture in the future, which is the clinical question that needs to be addressed and what PHASES was developed for. Practically compiling the necessary longitudinal datasets to answer this question is challenging, however, due to the relatively low short rates of rupture, which mean very large datasets with very long periods of follow up are necessary. This is now being addressed in the Risk of Aneurysm Rupture (ROAR) study which is following up the largest cohort of patients with unruptured aneurysms to date with more than double the cases of the whole PHASES metanalysis combined and far longer periods of follow up available [46]. Finally, our model was only validated internally, and we did not perform external validation. This is common to most computational studies [14–16, 21, 24], with the notable exception of [47]. The dataset used in this study, however, were collected during the @neurIST project which, despite being a large multicentric effort involving twenty-nine partners from twelve countries, did not have a uniformed data collection protocol. This implicitly guarantees that our model is robust to various images modalities. The size of the dataset we used, 170 patients, did not allow for further subdivisions based on the hospitals, while at the same time maintaining a meaningful sample size. Further validation of this model can be performed by resorting, for example, to larger datasets such as AneuX [48].

In conclusion, we showed that using additional data derived from CFD and morphological analysis increases the ability of logistic regression models in separating ruptured aneurysms from unruptured ones using clinical variables alone. The resulting logistic regression models achieved AUC = 0.71 and used a reduced number of features which were obtained through semi-automated processing of medical images and CFD results. This approach has the potential to be included within current clinical protocols, once being extended and validated using longitudinal data and larger patient cohorts.

## Supplementary Information

Below is the link to the electronic supplementary material.

Supplementary file 1 (DOCX 41 KB)

## Data Availability

Restrictions apply to the availability of patients’ data, either clinical or derived from computational fluid dynamics and shape analysis and are not publicly available. Contact IB (i.benemerito@sheffield.ac.uk) for collaboration requests.
