# Supplementary material for: Computational fluid dynamics and shape analysis enhance aneurysm rupture risk stratification
Source: Int J Comput Assist Radiol Surg. 2024 Nov 17;20(1):31–41. doi: 10.1007/s11548-024-03289-7 (PMC11757871; doi:10.1007/s11548-024-03289-7)
Supplement: Supplementary file 1 — (DOCX 41 KB) [file 11548_2024_3289_MOESM1_ESM.docx]

Supplementary material for:

**Computational fluid dynamics and shape analysis enhance aneurysm rupture risk stratification**

Ivan Benemerito^1,2^, Frederick Ewbank^3^, Andrew Narracott^1,4^, Maria-Cruz Villa-Uriol^1,5^, Ana Paula Narata^6^, Umang Patel^7^, Diederik Bulters^3^, Alberto Marzo^1,2^

^1^ INSIGNEO Institute for in silico medicine, University of Sheffield, Sheffield, UK

^2^ Department of Mechanical Engineering, University of Sheffield, Sheffield, UK

^3^ Department of Neurosurgery, University Hospital Southampton, Southampton, UK

^4^ Department of Neuroradiology, University Hospital Southampton, Southampton, UK

^5^ Department of Computer Science, University of Sheffield, Sheffield, UK

^6^ Department of Neuroradiology, University Hospital Southampton, Southampton, UK

^7^ Department of Neurosurgery, Oxford University Hospital NHS Foundation Trust, Oxford, UK

**Use of aggregated PHASES score to stratify ruptured aneurysms**

The same procedure described in the Methodology section of the paper was followed to develop a logistic regression model for aneurysm rupture that uses as predictor the aggregated PHASES score rather than its individual components. A 80/20 training/validation split was used to train the ridge constrained logistic regression model, with ten-fold cross validation and 500 bootstrap resampling iterations.

The model achieved an AUC = 55.8±8.9, a worse performance than all the other cases. The coefficients of the final logistic regression model and their 95% confidence intervals are:

| Feature | Logistic regression coefficients |
| --- | --- |
| PHASES score | 0.073 [0.073, 0.076] |
| Intercept | -0.295 [-0.306,-0.284] |

This logistic regression model performs worse than the five models reported in the manuscript. This is not unexpected since, as the reviewer pointed out, the PHASES score was developed on a dataset with a different ruptured/unruptured ratio and adopting longitudinal rather than cross-sectional data. A further reason is the distribution of the aggregate PHASES score in the unruptured and ruptured group, shown in the figure below:


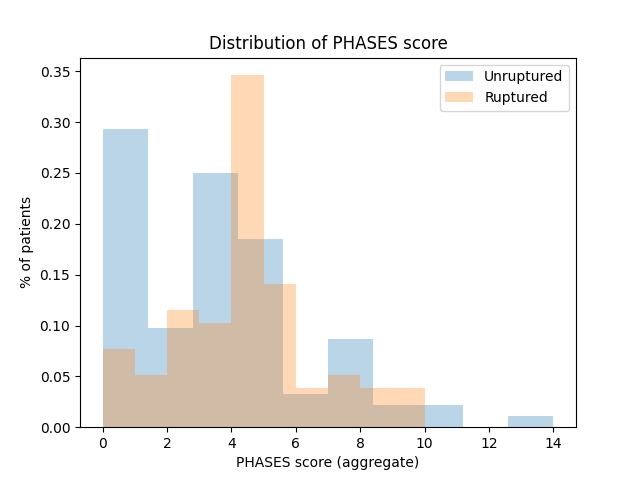


The degree of overlap between the two distributions is significant, implying that a regressor that is based exclusively based on this feature will have a limited stratification power.
